# Supplementary material for: The Role of Cytokines in the Metastasis of Solid Tumors to the Spine: Systematic Review
Source: Int J Mol Sci. 2023 Feb 14;24(4):3785. doi: 10.3390/ijms24043785 (PMC9962202; doi:10.3390/ijms24043785)
Supplement: Supplementary file 1 [file ijms-24-03785-s001.zip › ijms-2125077-supplementary.pdf]

Supplementary Table S1. List of cytokines and cytokine receptors play a role in bone metastases.

Cytokine, receptor	Primary tumor	Reference	Notes
**BFGF**	Breast	Meng X. *et al.* Myeloid-specific TGF-beta signaling in bone promotes basic-FGF and breast cancer bone metastasis. *Oncogene* **2016**, *35*, 2370-2378, doi:10.1038/onc.2015.297.	
	Prostate	Meng X. *et al.* Loss of TGF-beta signaling in osteoblasts increases basic-FGF and promotes prostate cancer bone metastasis. *Cancer Lett* **2018**, *418*, 109-118, doi:10.1016/j.canlet.2018.01.018.	
**BMP**	Prostate	Straign D.M. *et al.* Targeting the BMP Pathway in Prostate Cancer Induced Bone Disease. *Front Endocrinol (Lausanne)* **2021**, *12*, 769316, doi:10.3389/fendo.2021.769316.	
**BMP2**	Breast	Ye S. *et al.* In vivo inhibition of bone morphogenetic protein-2 on breast cancer cell growth. *Spine (Phila Pa 1976)* **2013**, *38*, E143-150, doi:10.1097/BRS.0b013e31827db4c6.	
	Breast	Moreau J.E. *et al.* Tissue-engineered bone serves as a target for metastasis of human breast cancer in a mouse model. *Cancer Res* **2007**, *67*, 10304-10308, doi:10.1158/0008-5472.CAN-07-2483.	
	Breast	Halpern J. *et al.* The application of a murine bone bioreactor as a model of tumor: bone interaction. *Clin Exp Metastasis* **2006**, *23*, 345-356, doi:10.1007/s10585-006-9044-8.	
	Prostate	Feeley B.T. *et al.* Overexpression of noggin inhibits BMP-mediated growth of osteolytic prostate cancer lesions. *Bone* **2006**, *38*, 154-166, doi:10.1016/j.bone.2005.07.015.	
**BMP7**	Breast	Buijs J.T. *et al.* Bone morphogenetic protein 7 in the development and treatment of bone metastases from breast cancer. *Cancer Res* **2007**, *67*, 8742-8751, doi:10.1158/0008-5472.CAN-06-2490.	
**BMPR1a**	Breast	Liu Y. *et al.* Knockdown of Bone Morphogenetic Proteins Type 1a Receptor (BMPR1a) in Breast Cancer Cells Protects Bone from Breast Cancer-Induced Osteolysis by Suppressing RANKL Expression. *Cell Physiol Biochem* **2018**, *45*, 1759-1771, doi:10.1159/000487784.	
**BSP**	Breast	Wang L. *et al.* Bone sialoprotein-alphavbeta3 integrin axis promotes breast cancer metastasis to the bone. *Cancer Sci* **2019**, *110*, 3157-3172, doi:10.1111/cas.14172.	
	Breast	Reufsteck C. *et al.* Silencing of skeletal metastasis-associated genes impairs migration of breast cancer cells and reduces osteolytic bone lesions. *Clin Exp Metastasis* **2012**, *29*, 441-456, doi:10.1007/s10585-012-9462-8.	
	Breast	Elazar V. *et al.* Sustained delivery and efficacy of polymeric nanoparticles containing osteopontin and bone sialoprotein antisenses in rats with breast cancer bone metastasis. *Int J Cancer* **2010**, *126*, 1749-1760, doi:10.1002/ijc.24890.	
	Breast	Adwan H. *et al.* Downregulation of osteopontin and bone sialoprotein II is related to reduced colony formation and metastasis formation of MDA-MB-231 human breast cancer cells. *Cancer Gene Ther* **2004**, *11*, 109-120, doi:10.1038/sj.cgt.7700659.	

**CCL2**	Breast	Takahashi M. *et al.* Chemokine CCL2/MCP-1 negatively regulates metastasis in a highly bone marrow-metastatic mouse breast cancer model. *Clin Exp Metastasis* **2009**, *26*, 817-828, doi:10.1007/s10585-009-9281-8.	Refers to spine metastases
	Breast	Bonapace L. *et al.* Cessation of CCL2 inhibition accelerates breast cancer metastasis by promoting angiogenesis. *Nature* **2014**, *515*, 130-133, doi:10.1038/nature13862.	
	Prostate	Park S.I. *et al.* Cyclophosphamide creates a receptive microenvironment for prostate cancer skeletal metastasis. *Cancer Res* **2012**, *72*, 2522-2532, doi:10.1158/0008-5472.CAN-11-2928.	
	Prostate	Mizutani K. *et al.* The chemokine CCL2 increases prostate tumor growth and bone metastasis through macrophage and osteoclast recruitment. *Neoplasia* **2009**, *11*, 1235-1242, doi:10.1593/neo.09988.	
	Prostate	Li X. *et al.* A destructive cascade mediated by CCL2 facilitates prostate cancer growth in bone. *Cancer Res* **2009**, *69*, 1685-1692, doi:10.1158/0008-5472.CAN-08-2164.	
	Prostate	Lu Y. *et al.* Activation of MCP-1/CCR2 axis promotes prostate cancer growth in bone. *Clin Exp Metastasis* **2009**, *26*, 161-169, doi:10.1007/s10585-008-9226-7.	
**CCL20**	Breast	Lee S.K. *et al.* Human antigen R-regulated CCL20 contributes to osteolytic breast cancer bone metastasis. *Sci Rep* **2017**, *7*, 9610, doi:10.1038/s41598-017-09040-4.	
	Prostate	Kfoury Y. *et al.* Human prostate cancer bone metastases have an actionable immunosuppressive microenvironment. *Cancer Cell* **2021**, *39*, 1464-1478 e1468, doi:10.1016/j.ccell.2021.09.005.	
**CCL21**	Breast	Weitzenfeld P. *et al.* Chemokine axes in breast cancer: factors of the tumor microenvironment reshape the CCR7-driven metastatic spread of luminal-A breast tumors. *J Leukoc Biol* **2016**, *99*, 1009-1025, doi:10.1189/jlb.3MA0815-373R.	
**CCL3**	Breast	Zuo H. *et al.* Differential regulation of breast cancer bone metastasis by PARP1 and PARP2. *Nat Commun* **2020**, *11*, 1578, doi:10.1038/s41467-020-15429-z.	
	Colon/ rectum	Zi-Chen G. *et al.* Colorectal cancer cells promote osteoclastogenesis and bone destruction through regulating EGF/ERK/CCL3 pathway. *Biosci Rep* **2020**, *40*, doi:10.1042/BSR20201175.	
**CCL4**	Breast	Sasaki S. *et al.* Essential roles of the interaction between cancer cell-derived chemokine, CCL4, and intra-bone CCR5-expressing fibroblasts in breast cancer bone metastasis. *Cancer Lett* **2016**, *378*, 23-32, doi:10.1016/j.canlet.2016.05.005.	
**CCL5**	Prostate	Huang R. *et al.* CCL5 derived from tumor-associated macrophages promotes prostate cancer stem cells and metastasis via activating beta-catenin/STAT3 signaling. *Cell Death Dis* **2020**, *11*, 234, doi:10.1038/s41419-020-2435-y.	
**CX3CL1**	Breast	Jamieson-Gladney W.L. *et al.* The chemokine receptor CX(3)CR1 is directly involved in the arrest of breast cancer cells to the skeleton. *Breast Cancer Res* **2011**, *13*, R91, doi:10.1186/bcr3016.	
	Liver	Sun C. *et al.* ADAM17-regulated CX3CL1 expression produced by bone marrow endothelial cells promotes spinal metastasis from hepatocellular carcinoma. *Int J Oncol* **2020**, *57*, 249-263, doi:10.3892/ijo.2020.5045.	Refers to spine metastases
**CX3CR1**	Liver	Sun C., Hu, A., Wang, S., Tian, B., Jiang, L., Liang, Y., Wang, H. and Dong, J. ADAM17-regulated CX3CL1 expression produced by bone marrow endothelial cells promotes spinal metastasis from hepatocellular carcinoma. *Int J Oncol* **2020**, *57*, 249-263, doi:10.3892/ijo.2020.5045.	Refers to spine metastases

**CXCL10**	Breast	Lee J.H. *et al.* CXCL10 promotes osteolytic bone metastasis by enhancing cancer outgrowth and osteoclastogenesis. *Cancer Res* **2012**, *72*, 3175-3186, doi:10.1158/0008-5472.CAN-12-0481.	
	Skin	Lee J.H., Kim, H.N., Kim, K.O., Jin, W.J., Lee, S., Kim, H.H., Ha, H. and Lee, Z.H. CXCL10 promotes osteolytic bone metastasis by enhancing cancer outgrowth and osteoclastogenesis. *Cancer Res* **2012**, *72*, 3175-3186, doi:10.1158/0008-5472.CAN-12-0481.	
**CXCL12**	Breast	Hang L.H. *et al.* Connexin 43 Mediates CXCL12 Production from Spinal Dorsal Horn to Maintain Bone Cancer Pain in Rats. *Neurochem Res* **2016**, *41*, 1200-1208, doi:10.1007/s11064-015-1815-7.	
	Breast	Zhang X.H. *et al.* Selection of bone metastasis seeds by mesenchymal signals in the primary tumor stroma. *Cell* **2013**, *154*, 1060-1073, doi:10.1016/j.cell.2013.07.036.	
	Prostate	Huang Z. *et al.* beta2AR-HIF-1alpha-CXCL12 signaling of osteoblasts activated by isoproterenol promotes migration and invasion of prostate cancer cells. *BMC Cancer* **2019**, *19*, 1142, doi:10.1186/s12885-019-6301-1.	
	Prostate	Osawa T. *et al.* Macrofluidic recirculating model of skeletal metastasis. *Sci Rep* **2019**, *9*, 14979, doi:10.1038/s41598-019-50577-3.	
	Prostate	Dai J. *et al.* Primary prostate cancer educates bone stroma through exosomal pyruvate kinase M2 to promote bone metastasis. *J Exp Med* **2019**, *216*, 2883-2899, doi:10.1084/jem.20190158.	
	Skin	Mannavola F. *et al.* Tumor-derived exosomes promote the in vitro osteotropism of melanoma cells by activating the SDF-1/CXCR4/CXCR7 axis. *J Transl Med* **2019**, *17*, 230, doi:10.1186/s12967-019-1982-4.	
**CXCL12G**	Breast	Ray P. *et al.* CXCL12-gamma in primary tumors drives breast cancer metastasis. *Oncogene* **2015**, *34*, 2043-2051, doi:10.1038/onc.2014.157.	
	Prostate	Jung Y. *et al.* CXCL12gamma Promotes Metastatic Castration-Resistant Prostate Cancer by Inducing Cancer Stem Cell and Neuroendocrine Phenotypes. *Cancer Res* **2018**, *78*, 2026-2039, doi:10.1158/0008-5472.CAN-17-2332.	
**CXCL16**	Lung	Ajona D. *et al.* Blockade of the Complement C5a/C5aR1 Axis Impairs Lung Cancer Bone Metastasis by CXCL16-mediated Effects. *Am J Respir Crit Care Med* **2018**, *197*, 1164-1176, doi:10.1164/rccm.201703-0660OC.	
**CXCL5**	Prostate	Roca H. *et al.* Apoptosis-induced CXCL5 accelerates inflammation and growth of prostate tumor metastases in bone. *J Clin Invest* **2018**, *128*, 248-266, doi:10.1172/JCI92466.	Refers to spine metastases
**CXCR3**	Breast	Jin W.J. *et al.* NF-kappaB signaling regulates cell-autonomous regulation of CXCL10 in breast cancer 4T1 cells. *Exp Mol Med* **2017**, *49*, e295, doi:10.1038/emm.2016.148.	
**CXCR4**	Breast	Price T.T. *et al.* Dormant breast cancer micrometastases reside in specific bone marrow niches that regulate their transit to and from bone. *Sci Transl Med* **2016**, *8*, 340ra373, doi:10.1126/scitranslmed.aad4059.	
	Breast	Xiang J. *et al.* CXCR4 Protein Epitope Mimetic Antagonist POL5551 Disrupts Metastasis and Enhances Chemotherapy Effect in Triple-Negative Breast Cancer. *Mol Cancer Ther* **2015**, *14*, 2473-2485, doi:10.1158/1535-7163.MCT-15-0252.	
	Lung	Ma N. *et al.* Downregulation of CXCR4 by SDF-KDEL in SBC-5 cells inhibits their migration in vitro and organ metastasis in vivo. *Int J Mol Med* **2015**, *35*, 425-432, doi:10.3892/ijmm.2014.2033.	
	Nervous system	Muhlethaler-Mottet A. *et al.* The CXCR4/CXCR7/CXCL12 Axis Is Involved in a Secondary but Complex Control of Neuroblastoma Metastatic Cell Homing. *PLoS One* **2015**, *10*, e0125616, doi:10.1371/journal.pone.0125616.	

	Nervous system	Zhang L. *et al.* Tissue microenvironment modulates CXCR4 expression and tumor metastasis in neuroblastoma. *Neoplasia* **2007**, *9*, 36-46, doi:10.1593/neo.06670.	
	Prostate	Jung Y. *et al.* Recruitment of mesenchymal stem cells into prostate tumours promotes metastasis. *Nat Commun* **2013**, *4*, 1795, doi:10.1038/ncomms2766.	Refers to spine metastases
	Prostate	Zhang B. *et al.* Acetylation of KLF5 maintains EMT and tumorigenicity to cause chemoresistant bone metastasis in prostate cancer. *Nat Commun* **2021**, *12*, 1714, doi:10.1038/s41467-021-21976-w.	
	Prostate	Conley-LaComb M.K. *et al.* Pharmacological targeting of CXCL12/CXCR4 signaling in prostate cancer bone metastasis. *Mol Cancer* **2016**, *15*, 68, doi:10.1186/s12943-016-0552-0.	
	Prostate	Gravina G.L. *et al.* CXCR4 pharmacogical inhibition reduces bone and soft tissue metastatic burden by affecting tumor growth and tumorigenic potential in prostate cancer preclinical models. *Prostate* **2015**, *75*, 1227-1246, doi:10.1002/pros.23007.	
	Prostate	Wang N. *et al.* Prostate cancer cells preferentially home to osteoblast-rich areas in the early stages of bone metastasis: evidence from in vivo models. *J Bone Miner Res* **2014**, *29*, 2688-2696, doi:10.1002/jbmr.2300.	
	Prostate	Xing Y. *et al.* Tumor cell-specific blockade of CXCR4/SDF-1 interactions in prostate cancer cells by hTERT promoter induced CXCR4 knockdown: A possible metastasis preventing and minimizing approach. *Cancer Biol Ther* **2008**, *7*, 1839-1848, doi:10.4161/cbt.7.11.6862.	
	Prostate	Chinni S.R. *et al.* CXCL12/CXCR4 transactivates HER2 in lipid rafts of prostate cancer cells and promotes growth of metastatic deposits in bone. *Mol Cancer Res* **2008**, *6*, 446-457, doi:10.1158/1541-7786.MCR-07-0117.	
	Prostate	Sun Y.X. *et al.* Skeletal localization and neutralization of the SDF-1(CXCL12)/CXCR4 axis blocks prostate cancer metastasis and growth in osseous sites in vivo. *J Bone Miner Res* **2005**, *20*, 318-329, doi:10.1359/JBMR.041109.	
**CXCR7**	Nervous system	Muhlethaler-Mottet A., Liberman, J., Ascencao, K., Flahaut, M., Balmas Bourloud, K., Yan, P., Jauquier, N., Gross, N. and Joseph, J.M. The CXCR4/CXCR7/CXCL12 Axis Is Involved in a Secondary but Complex Control of Neuroblastoma Metastatic Cell Homing. *PLoS One* **2015**, *10*, e0125616, doi:10.1371/journal.pone.0125616.	
**EGFR**	Breast	Lu X. *et al.* ADAMTS1 and MMP1 proteolytically engage EGF-like ligands in an osteolytic signaling cascade for bone metastasis. *Genes Dev* **2009**, *23*, 1882-1894, doi:10.1101/gad.1824809.	
	Prostate	Li Y. *et al.* Antitumor and antimetastatic activities of docetaxel are enhanced by genistein through regulation of osteoprotegerin/receptor activator of nuclear factor-kappaB (RANK)/RANK ligand/MMP-9 signaling in prostate cancer. *Cancer Res* **2006**, *66*, 4816-4825, doi:10.1158/0008-5472.CAN-05-3752.	
	Prostate	Kim S.J. *et al.* Blockade of epidermal growth factor receptor signaling in tumor cells and tumor-associated endothelial cells for therapy of androgen-independent human prostate cancer growing in the bone of nude mice. *Clin Cancer Res* **2003**, *9*, 1200-1210, .	
	Vulva	Canon J. *et al.* Inhibition of RANKL increases the anti-tumor effect of the EGFR inhibitor panitumumab in a murine model of bone metastasis. *Bone* **2010**, *46*, 1613-1619, doi:10.1016/j.bone.2010.03.001.	
**FGFR**	Breast	Kang J. *et al.* A Selective FGFR inhibitor AZD4547 suppresses RANKL/M-CSF/OPG-dependent ostoclastogenesis and breast cancer growth in the metastatic bone microenvironment. *Sci Rep* **2019**, *9*, 8726, doi:10.1038/s41598-019-45278-w.	

**G-CSF**	Breast	Hiraga T. *et al.* Opposing Effects of Granulocyte Colony-Stimulating Factor on the Initiation and Progression of Breast Cancer Bone Metastases. *Mol Cancer Res* **2021**, *19*, 2110-2119, doi:10.1158/1541-7786.MCR-21-0243.
	Breast	Yip R.K.H. *et al.* Mammary tumour cells remodel the bone marrow vascular microenvironment to support metastasis. *Nat Commun* **2021**, *12*, 6920, doi:10.1038/s41467-021-26556-6.
**GDF15**	Prostate	Wang W. *et al.* Prostate cancer promotes a vicious cycle of bone metastasis progression through inducing osteocytes to secrete GDF15 that stimulates prostate cancer growth and invasion. *Oncogene* **2019**, *38*, 4540-4559, doi:10.1038/s41388-019-0736-3.
	Prostate	Wakchoure S. *et al.* Expression of macrophage inhibitory cytokine-1 in prostate cancer bone metastases induces osteoclast activation and weight loss. *Prostate* **2009**, *69*, 652-661, doi:10.1002/pros.20913.
**GM-CSF**	Breast	Dai J. *et al.* Reversal of chemotherapy-induced leukopenia using granulocyte macrophage colony-stimulating factor promotes bone metastasis that can be blocked with osteoclast inhibitors. *Cancer Res* **2010**, *70*, 5014-5023, doi:10.1158/0008-5472.CAN-10-0100.
	Breast	Park B.K. *et al.* NF-kappaB in breast cancer cells promotes osteolytic bone metastasis by inducing osteoclastogenesis via GM-CSF. *Nat Med* **2007**, *13*, 62-69, doi:10.1038/nm1519.
	Prostate	Dai J., Lu, Y., Yu, C., Keller, J.M., Mizokami, A., Zhang, J. and Keller, E.T. Reversal of chemotherapy-induced leukopenia using granulocyte macrophage colony-stimulating factor promotes bone metastasis that can be blocked with osteoclast inhibitors. *Cancer Res* **2010**, *70*, 5014-5023, doi:10.1158/0008-5472.CAN-10-0100.
	Prostate	Small E.J. *et al.* Granulocyte macrophage colony-stimulating factor--secreting allogeneic cellular immunotherapy for hormone-refractory prostate cancer. *Clin Cancer Res* **2007**, *13*, 3883-3891, doi:10.1158/1078-0432.CCR-06-2937.
**HGF**	Breast	Yao Y. *et al.* HGFK1 inhibits bone metastasis in breast cancer through the TAK1/p38 MAPK signaling pathway. *Cancer Gene Ther* **2012**, *19*, 601-608, doi:10.1038/cgt.2012.38.
	Lung	Navab R. *et al.* Co-overexpression of Met and hepatocyte growth factor promotes systemic metastasis in NCI-H460 non-small cell lung carcinoma cells. *Neoplasia* **2009**, *11*, 1292-1300, doi:10.1593/neo.09622.
**HGFR**	Lung	Fujita H. *et al.* High Potency VEGFRs/MET/FMS Triple Blockade by TAS-115 Concomitantly Suppresses Tumor Progression and Bone Destruction in Tumor-Induced Bone Disease Model with Lung Carcinoma Cells. *PLoS One* **2016**, *11*, e0164830, doi:10.1371/journal.pone.0164830.
**IBSP**	Breast	Wu K. *et al.* Exosomal miR-19a and IBSP cooperate to induce osteolytic bone metastasis of estrogen receptor-positive breast cancer. *Nat Commun* **2021**, *12*, 5196, doi:10.1038/s41467-021-25473-y.
**IFN**	Breast	Bidwell B.N. *et al.* Silencing of Irf7 pathways in breast cancer cells promotes bone metastasis through immune escape. *Nat Med* **2012**, *18*, 1224-1231, doi:10.1038/nm.2830.
**IFNA**	Kidney	Kurabayashi A. *et al.* Combination with third-generation bisphosphonate (YM529) and interferon-alpha can inhibit the progression of established bone renal cell carcinoma. *Cancer Sci* **2015**, *106*, 1092-1099, doi:10.1111/cas.12711.
**IFNAR1**	Breast	Rautela J. *et al.* Loss of Host Type-I IFN Signaling Accelerates Metastasis and Impairs NK-cell Antitumor Function in Multiple Models of Breast Cancer. *Cancer Immunol Res* **2015**, *3*, 1207-1217, doi:10.1158/2326-6066.CIR-15-0065.
**IFNG**	Lung	Young M.R. and Wright, M.A. Myelopoiesis-associated immune suppressor cells in mice bearing metastatic Lewis lung carcinoma tumors: gamma interferon plus tumor necrosis factor alpha synergistically reduces immune suppressor and

		tumor growth-promoting activities of bone marrow cells and diminishes tumor recurrence and metastasis. *Cancer Res* **1992**, *52*, 6335-6340, .	
**IGF1**	Breast	Zhang X.H., Jin, X., Malladi, S., Zou, Y., Wen, Y.H., Brogi, E., Smid, M., Foekens, J.A. and Massague, J. Selection of bone metastasis seeds by mesenchymal signals in the primary tumor stroma. *Cell* **2013**, *154*, 1060-1073, doi:10.1016/j.cell.2013.07.036.	
**IL1**	Skin	Anasagasti M.J. *et al.* Interleukin 1-dependent and -independent mouse melanoma metastases. *J Natl Cancer Inst* **1997**, *89*, 645-651, doi:10.1093/jnci/89.9.645.	
	Skin	Arguello F. *et al.* Effect of IL-1 on experimental bone/bone-marrow metastases. *Int J Cancer* **1992**, *52*, 802-807, doi:10.1002/ijc.2910520522.	
**IL10**	Prostate	Stearns M.E. and Wang, M. Antimestatic and antitumor activities of interleukin 10 in transfected human prostate PC-3 ML clones: Orthotopic growth in severe combined immunodeficient mice. *Clin Cancer Res* **1998**, *4*, 2257-2263, .	Refers to spine metastases
**IL12**	Brain	Lode H.N. *et al.* Gene therapy with a single chain interleukin 12 fusion protein induces T cell-dependent protective immunity in a syngeneic model of murine neuroblastoma. *Proc Natl Acad Sci U S A* **1998**, *95*, 2475-2480, doi:10.1073/pnas.95.5.2475.	
	Prostate	Wang H. *et al.* IL-12 gene-modified bone marrow cell therapy suppresses the development of experimental metastatic prostate cancer. *Cancer Gene Ther* **2007**, *14*, 819-827, doi:10.1038/sj.cgt.7701069.	
**IL17**	Breast	Roy L.D. *et al.* Collagen induced arthritis increases secondary metastasis in MMTV-PyV MT mouse model of mammary cancer. *BMC Cancer* **2011**, *11*, 365, doi:10.1186/1471-2407-11-365.	
**IL17A**	Breast	Roy L.D. *et al.* Systemic neutralization of IL-17A significantly reduces breast cancer associated metastasis in arthritic mice by reducing CXCL12/SDF-1 expression in the metastatic niches. *BMC Cancer* **2014**, *14*, 225, doi:10.1186/1471-2407-14-225.	
**IL18**	Breast	Nakata A. *et al.* Inhibition by interleukin 18 of osteolytic bone metastasis by human breast cancer cells. *Anticancer Res* **1999**, *19*, 4131-4138, .	
	Lung	Iwasaki T. *et al.* Interleukin-18 inhibits osteolytic bone metastasis by human lung cancer cells possibly through suppression of osteoclastic bone-resorption in nude mice. *J Immunother* **2002**, *25 Suppl 1*, S52-60, doi:10.1097/00002371-200203001-00008.	
**IL1B**	Breast	Tulotta C. *et al.* IL-1B drives opposing responses in primary tumours and bone metastases; harnessing combination therapies to improve outcome in breast cancer. *NPJ Breast Cancer* **2021**, *7*, 95, doi:10.1038/s41523-021-00305-w.	
	Breast	Eyre R. *et al.* Microenvironmental IL1beta promotes breast cancer metastatic colonisation in the bone via activation of Wnt signalling. *Nat Commun* **2019**, *10*, 5016, doi:10.1038/s41467-019-12807-0.	
	Breast	Tulotta C. *et al.* Endogenous Production of IL1B by Breast Cancer Cells Drives Metastasis and Colonization of the Bone Microenvironment. *Clin Cancer Res* **2019**, *25*, 2769-2782, doi:10.1158/1078-0432.CCR-18-2202.	
	Breast	Holen I. *et al.* IL-1 drives breast cancer growth and bone metastasis in vivo. *Oncotarget* **2016**, *7*, 75571-75584, doi:10.18632/oncotarget.12289.	
	Prostate	Shahriari K. *et al.* Cooperation among heterogeneous prostate cancer cells in the bone metastatic niche. *Oncogene* **2017**, *36*, 2846-2856, doi:10.1038/onc.2016.436.	

**IL2**	Nervous system	Salcedo R. *et al.* Immunologic and therapeutic synergy of IL-27 and IL-2: enhancement of T cell sensitization, tumor-specific CTL reactivity and complete regression of disseminated neuroblastoma metastases in the liver and bone marrow. *J Immunol* **2009**, *182*, 4328-4338, doi:10.4049/jimmunol.0800471.
	Nervous system	Lode H.N. *et al.* Targeted interleukin-2 therapy for spontaneous neuroblastoma metastases to bone marrow. *J Natl Cancer Inst* **1997**, *89*, 1586-1594, doi:10.1093/jnci/89.21.1586.
	Skin	Quan W.D., Jr. *et al.* Outpatient intravenous interleukin-2 with famotidine has activity in metastatic melanoma. *Cancer Biother Radiopharm* **2012**, *27*, 442-445, doi:10.1089/cbr.2012.1239.
**IL20**	Breast	Hsu Y.H. *et al.* Anti-IL-20 monoclonal antibody suppresses breast cancer progression and bone osteolysis in murine models. *J Immunol* **2012**, *188*, 1981-1991, doi:10.4049/jimmunol.1102843.
**IL24**	Prostate	Pradhan A.K. *et al.* Recombinant MDA-7/IL24 Suppresses Prostate Cancer Bone Metastasis through Downregulation of the Akt/Mcl-1 Pathway. *Mol Cancer Ther* **2018**, *17*, 1951-1960, doi:10.1158/1535-7163.MCT-17-1002.
**IL27**	Nervous system	Salcedo R., Hixon, J.A., Stauffer, J.K., Jalah, R., Brooks, A.D., Khan, T., Dai, R.M., Scheetz, L., Lincoln, E., Back, T.C., Powell, D., Hurwitz, A.A., Sayers, T.J., Kastelein, R., Pavlakis, G.N., Felber, B.K., Trinchieri, G. and Wigginton, J.M. Immunologic and therapeutic synergy of IL-27 and IL-2: enhancement of T cell sensitization, tumor-specific CTL reactivity and complete regression of disseminated neuroblastoma metastases in the liver and bone marrow. *J Immunol* **2009**, *182*, 4328-4338, doi:10.4049/jimmunol.0800471.
**IL4**	Breast	Lei W. *et al.* The IAP Antagonist SM-164 Eliminates Triple-Negative Breast Cancer Metastasis to Bone and Lung in Mice. *Sci Rep* **2020**, *10*, 7004, doi:10.1038/s41598-020-64018-z.
**IL6**	Breast	Remeniuk B. *et al.* Disease modifying actions of interleukin-6 blockade in a rat model of bone cancer pain. *Pain* **2018**, *159*, 684-698, doi:10.1097/j.pain.0000000000001139.
	Breast	Wakabayashi H. *et al.* Interleukin-6 receptor inhibitor suppresses bone metastases in a breast cancer cell line. *Breast Cancer* **2018**, *25*, 566-574, doi:10.1007/s12282-018-0853-9.
	Breast	Luo X. *et al.* Stromal-Initiated Changes in the Bone Promote Metastatic Niche Development. *Cell Rep* **2016**, *14*, 82-92, doi:10.1016/j.celrep.2015.12.016.
	Breast	Zheng Y. *et al.* Direct crosstalk between cancer and osteoblast lineage cells fuels metastatic growth in bone via auto-amplification of IL-6 and RANKL signaling pathways. *J Bone Miner Res* **2014**, *29*, 1938-1949, doi:10.1002/jbmr.2231.
	Pancreas	Grunwald B. *et al.* Systemic Ablation of MMP-9 Triggers Invasive Growth and Metastasis of Pancreatic Cancer via Deregulation of IL6 Expression in the Bone Marrow. *Mol Cancer Res* **2016**, *14*, 1147-1158, doi:10.1158/1541-7786.MCR-16-0180.
	Prostate	Zheng Y. *et al.* Targeting IL-6 and RANKL signaling inhibits prostate cancer growth in bone. *Clin Exp Metastasis* **2014**, *31*, 921-933, doi:10.1007/s10585-014-9680-3.
	Prostate	Kim S.W. *et al.* Consistent interactions between tumor cell IL-6 and macrophage TNF-alpha enhance the growth of human prostate cancer cells in the bone of nude mouse. *Int Immunopharmacol* **2011**, *11*, 862-872, doi:10.1016/j.intimp.2011.01.004.
**IL7**	Prostate	Seol M.A. *et al.* Interleukin-7 Contributes to the Invasiveness of Prostate Cancer Cells by Promoting Epithelial-Mesenchymal Transition. *Sci Rep* **2019**, *9*, 6917, doi:10.1038/s41598-019-43294-4.

**IL8**	Breast	Kamalakar A. *et al.* Circulating interleukin-8 levels explain breast cancer osteolysis in mice and humans. *Bone* **2014**, *61*, 176-185, doi:10.1016/j.bone.2014.01.015.
	Lung	Iguchi H. *et al.* Overproduction of IL-8 results in suppression of bone metastasis by lung cancer cells in vivo. *Int J Oncol* **2000**, *17*, 329-333, doi:10.3892/ijo.17.2.329.
**LIF**	Breast	Johnson R.W. *et al.* Induction of LIFR confers a dormancy phenotype in breast cancer cells disseminated to the bone marrow. *Nat Cell Biol* **2016**, *18*, 1078-1089, doi:10.1038/ncb3408.
	Muscle	Wysoczynski M. *et al.* Leukemia inhibitory factor: a newly identified metastatic factor in rhabdomyosarcomas. *Cancer Res* **2007**, *67*, 2131-2140, doi:10.1158/0008-5472.CAN-06-1021.
	Skin	Maruta S. *et al.* A role for leukemia inhibitory factor in melanoma-induced bone metastasis. *Clin Exp Metastasis* **2009**, *26*, 133-141, doi:10.1007/s10585-008-9223-x.
**M-CSF**	Breast	Jeffery J.J. *et al.* Autocrine inhibition of the c-fms proto-oncogene reduces breast cancer bone metastasis assessed with in vivo dual-modality imaging. *Exp Biol Med (Maywood)* **2014**, *239*, 404-413, doi:10.1177/1535370214522588.
	Lung	Hung J.Y. *et al.* Colony-stimulating factor 1 potentiates lung cancer bone metastasis. *Lab Invest* **2014**, *94*, 371-381, doi:10.1038/labinvest.2014.1.
**MET**	Lung	Fujita H., Gomori, A., Fujioka, Y., Kataoka, Y., Tanaka, K., Hashimoto, A., Suzuki, T., Ito, K., Haruma, T., Yamamoto-Yokoi, H., Harada, N., Sakuragi, M., Oda, N., Matsuo, K., Inada, M. and Yonekura, K. High Potency VEGFRs/MET/FMS Triple Blockade by TAS-115 Concomitantly Suppresses Tumor Progression and Bone Destruction in Tumor-Induced Bone Disease Model with Lung Carcinoma Cells. *PLoS One* **2016**, *11*, e0164830, doi:10.1371/journal.pone.0164830.
**OPG**	Breast	Ottewell P.D. *et al.* OPG-Fc inhibits ovariectomy-induced growth of disseminated breast cancer cells in bone. *Int J Cancer* **2015**, *137*, 968-977, doi:10.1002/ijc.29439.
	Breast	Chanda D. *et al.* Systemic osteoprotegerin gene therapy restores tumor-induced bone loss in a therapeutic model of breast cancer bone metastasis. *Mol Ther* **2008**, *16*, 871-878, doi:10.1038/mt.2008.48.
	Breast	Roudier M.P. *et al.* Effects of the RANKL inhibitor, osteoprotegerin, on the pain and histopathology of bone cancer in rats. *Clin Exp Metastasis* **2006**, *23*, 167-175, doi:10.1007/s10585-006-9026-x.
	Lung	Miller R.E. *et al.* RANKL inhibition blocks osteolytic lesions and reduces skeletal tumor burden in models of non-small-cell lung cancer bone metastases. *J Thorac Oncol* **2014**, *9*, 345-354, doi:10.1097/JTO.0000000000000070.
	Prostate	Uehara H. *et al.* Induction of retinol-binding protein 4 and placenta-specific 8 expression in human prostate cancer cells remaining in bone following osteolytic tumor growth inhibition by osteoprotegerin. *Int J Oncol* **2013**, *43*, 365-374, doi:10.3892/ijo.2013.1954.
	Prostate	Corey E. *et al.* Osteoprotegerin in prostate cancer bone metastasis. *Cancer Res* **2005**, *65*, 1710-1718, doi:10.1158/0008-5472.CAN-04-2033.
	Prostate	Zhang J. *et al.* Osteoprotegerin inhibits prostate cancer-induced osteoclastogenesis and prevents prostate tumor growth in the bone. *J Clin Invest* **2001**, *107*, 1235-1244, doi:10.1172/JCI11685.
**OPN**	Breast	Kovacheva M. *et al.* Conditional Knockdown of Osteopontin Inhibits Breast Cancer Skeletal Metastasis. *Int J Mol Sci* **2019**, *20*, doi:10.3390/ijms20194918.
	Breast	Kuo M.C. *et al.* Cancer stemness in bone marrow micrometastases of human breast cancer. *Surgery* **2018**, *163*, 330-335, doi:10.1016/j.surg.2017.07.027.

	Breast	Reufsteck C., Lifshitz-Shovali, R., Zepp, M., Bauerle, T., Kubler, D., Golomb, G. and Berger, M.R. Silencing of skeletal metastasis-associated genes impairs migration of breast cancer cells and reduces osteolytic bone lesions. *Clin Exp Metastasis* **2012**, *29*, 441-456, doi:10.1007/s10585-012-9462-8.
	Breast	Elazar V., Adwan, H., Bauerle, T., Rohekar, K., Golomb, G. and Berger, M.R. Sustained delivery and efficacy of polymeric nanoparticles containing osteopontin and bone sialoprotein antisenses in rats with breast cancer bone metastasis. *Int J Cancer* **2010**, *126*, 1749-1760, doi:10.1002/ijc.24890.
	Breast	Adwan H., Bauerle, T.J. and Berger, M.R. Downregulation of osteopontin and bone sialoprotein II is related to reduced colony formation and metastasis formation of MDA-MB-231 human breast cancer cells. *Cancer Gene Ther* **2004**, *11*, 109-120, doi:10.1038/sj.cgt.7700659.
	Skin	Nemoto H. *et al.* Osteopontin deficiency reduces experimental tumor cell metastasis to bone and soft tissues. *J Bone Miner Res* **2001**, *16*, 652-659, doi:10.1359/jbmr.2001.16.4.652.
	Skin	Ohyama Y. *et al.* Osteopontin-deficiency suppresses growth of B16 melanoma cells implanted in bone and osteoclastogenesis in co-cultures. *J Bone Miner Res* **2004**, *19*, 1706-1711, doi:10.1359/jbmr.2004.19.10.1706.
**OSM**	Breast	Bolin C. *et al.* Oncostatin m promotes mammary tumor metastasis to bone and osteolytic bone degradation. *Genes Cancer* **2012**, *3*, 117-130, doi:10.1177/1947601912458284.
**PLGF**	Breast	Coenegrachts L. *et al.* Anti-placental growth factor reduces bone metastasis by blocking tumor cell engraftment and osteoclast differentiation. *Cancer Res* **2010**, *70*, 6537-6547, doi:10.1158/0008-5472.CAN-09-4092.
**PTHRP**	Breast	Saito H. *et al.* Humanized monoclonal antibody against parathyroid hormone-related protein suppresses osteolytic bone metastasis of human breast cancer cells derived from MDA-MB-231. *Anticancer Res* **2005**, *25*, 3817-3823, .
	Breast	Kakonen S.M. *et al.* Transforming growth factor-beta stimulates parathyroid hormone-related protein and osteolytic metastases via Smad and mitogen-activated protein kinase signaling pathways. *J Biol Chem* **2002**, *277*, 24571-24578, doi:10.1074/jbc.M202561200.
	Breast	Yin J.J. *et al.* TGF-beta signaling blockade inhibits PTHrP secretion by breast cancer cells and bone metastases development. *J Clin Invest* **1999**, *103*, 197-206, doi:10.1172/JCI3523.
	Breast	Guise T.A. Parathyroid hormone-related protein and bone metastases. *Cancer* **1997**, *80*, 1572-1580, doi:10.1002/(sici)1097-0142(19971015)80:8+<1572::aid-cncr7>3.3.co;2-d.
	Lung	Iguchi H. *et al.* An experimental model of bone metastasis by human lung cancer cells: the role of parathyroid hormone-related protein in bone metastasis. *Cancer Res* **1996**, *56*, 4040-4043, .
	Lung	Miki T. *et al.* Parathyroid hormone-related protein (PTHrP) is responsible for production of bone metastasis, but not visceral metastasis, by human small cell lung cancer SBC-5 cells in natural killer cell-depleted SCID mice. *Int J Cancer* **2004**, *108*, 511-515, doi:10.1002/ijc.11586.
	Prostate	Park S.I. and McCauley, L.K. Nuclear localization of parathyroid hormone-related peptide confers resistance to anoikis in prostate cancer cells. *Endocr Relat Cancer* **2012**, *19*, 243-254, doi:10.1530/ERC-11-0278.
	Skin	Mannavola F., Tucci, M., Felici, C., Passarelli, A., D'Oronzo, S. and Silvestris, F. Tumor-derived exosomes promote the in vitro osteotropism of melanoma cells by activating the SDF-1/CXCR4/CXCR7 axis. *J Transl Med* **2019**, *17*, 230, doi:10.1186/s12967-019-1982-4.

**RANKL**	Breast	Yue Z. *et al.* RSPO2 and RANKL signal through LGR4 to regulate osteoclastic premetastatic niche formation and bone metastasis. *J Clin Invest* **2022**, *132*, doi:10.1172/JCI144579.
	Breast	Kim B. *et al.* Immunization With RANKL Inhibits Osteolytic Bone Metastasis in Breast Cancer. *J Immunother* **2022**, *45*, 1-12, doi:10.1097/CJI.0000000000000393.
	Breast	Lei W., Duan, R., Li, J., Liu, X., Huston, A., Boyce, B.F. and Yao, Z. The IAP Antagonist SM-164 Eliminates Triple-Negative Breast Cancer Metastasis to Bone and Lung in Mice. *Sci Rep* **2020**, *10*, 7004, doi:10.1038/s41598-020-64018-z.
	Breast	Pantano F. *et al.* Dynamic changes of Receptor activator of nuclear factor-kappaB expression in Circulating Tumor Cells during Denosumab predict treatment effectiveness in Metastatic Breast Cancer. *Sci Rep* **2020**, *10*, 1288, doi:10.1038/s41598-020-58339-2.
	Breast	Asano T. *et al.* Soluble RANKL is physiologically dispensable but accelerates tumour metastasis to bone. *Nat Metab* **2019**, *1*, 868-875, doi:10.1038/s42255-019-0104-1.
	Breast	Futakuchi M. *et al.* Therapeutic and Preventive Effects of Osteoclastogenesis Inhibitory Factor on Osteolysis, Proliferation of Mammary Tumor Cell and Induction of Cancer Stem Cells in the Bone Microenvironment. *Int J Mol Sci* **2018**, *19*, doi:10.3390/ijms19030888.
	Breast	Ottewell P.D., Wang, N., Brown, H.K., Fowles, C.A., Croucher, P.I., Eaton, C.L. and Holen, I. OPG-Fc inhibits ovariectomy-induced growth of disseminated breast cancer cells in bone. *Int J Cancer* **2015**, *137*, 968-977, doi:10.1002/ijc.29439.
	Breast	Zheng Y., Chow, S.O., Boernert, K., Basel, D., Mikuscheva, A., Kim, S., Fong-Yee, C., Trivedi, T., Buttgereit, F., Sutherland, R.L., Dunstan, C.R., Zhou, H. and Seibel, M.J. Direct crosstalk between cancer and osteoblast lineage cells fuels metastatic growth in bone via auto-amplification of IL-6 and RANKL signaling pathways. *J Bone Miner Res* **2014**, *29*, 1938-1949, doi:10.1002/jbmr.2231.
	Breast	Blake M.L. *et al.* RANK expression on breast cancer cells promotes skeletal metastasis. *Clin Exp Metastasis* **2014**, *31*, 233-245, doi:10.1007/s10585-013-9624-3.
	Breast	Canon J. *et al.* RANKL inhibition combined with tamoxifen treatment increases anti-tumor efficacy and prevents tumor-induced bone destruction in an estrogen receptor-positive breast cancer bone metastasis model. *Breast Cancer Res Treat* **2012**, *135*, 771-780, doi:10.1007/s10549-012-2222-2.
	Breast	Dai J., Lu, Y., Yu, C., Keller, J.M., Mizokami, A., Zhang, J. and Keller, E.T. Reversal of chemotherapy-induced leukopenia using granulocyte macrophage colony-stimulating factor promotes bone metastasis that can be blocked with osteoclast inhibitors. *Cancer Res* **2010**, *70*, 5014-5023, doi:10.1158/0008-5472.CAN-10-0100.
	Breast	Holland P.M. *et al.* Combined therapy with the RANKL inhibitor RANK-Fc and rhApo2L/TRAIL/dulanermin reduces bone lesions and skeletal tumor burden in a model of breast cancer skeletal metastasis. *Cancer Biol Ther* **2010**, *9*, 539-550, doi:10.4161/cbt.9.7.11266.
	Breast	Nannuru K.C. *et al.* Enhanced expression and shedding of receptor activator of NF-kappaB ligand during tumor-bone interaction potentiates mammary tumor-induced osteolysis. *Clin Exp Metastasis* **2009**, *26*, 797-808, doi:10.1007/s10585-009-9279-2.
	Breast	Futakuchi M. *et al.* Transforming growth factor-beta signaling at the tumor-bone interface promotes mammary tumor growth and osteoclast activation. *Cancer Sci* **2009**, *100*, 71-81, doi:10.1111/j.1349-7006.2008.01012.x.

Breast	Canon J.R. *et al.* Inhibition of RANKL blocks skeletal tumor progression and improves survival in a mouse model of breast cancer bone metastasis. *Clin Exp Metastasis* **2008**, *25*, 119-129, doi:10.1007/s10585-007-9127-1.
Breast	Roudier M.P., Bain, S.D. and Dougall, W.C. Effects of the RANKL inhibitor, osteoprotegerin, on the pain and histopathology of bone cancer in rats. *Clin Exp Metastasis* **2006**, *23*, 167-175, doi:10.1007/s10585-006-9026-x.
Lung	Niu Y. *et al.* Intervention with the Bone-Associated Tumor Vicious Cycle through Dual-Protein Therapeutics for Treatment of Skeletal-Related Events and Bone Metastases. *ACS Nano* **2022**, *16*, 2209-2223, doi:10.1021/acsnano.1c08269.
Lung	Brunetti G. *et al.* LIGHT/TNFSF14 Promotes Osteolytic Bone Metastases in Non-small Cell Lung Cancer Patients. *J Bone Miner Res* **2020**, *35*, 671-680, doi:10.1002/jbmr.3942.
Lung	Feeley B.T. *et al.* Mixed metastatic lung cancer lesions in bone are inhibited by noggin overexpression and Rank:Fc administration. *J Bone Miner Res* **2006**, *21*, 1571-1580, doi:10.1359/jbmr.060706.
Prostate	Zheng Y., Basel, D., Chow, S.O., Fong-Yee, C., Kim, S., Buttgereit, F., Dunstan, C.R., Zhou, H. and Seibel, M.J. Targeting IL-6 and RANKL signaling inhibits prostate cancer growth in bone. *Clin Exp Metastasis* **2014**, *31*, 921-933, doi:10.1007/s10585-014-9680-3.
Prostate	Chu G.C. *et al.* RANK- and c-Met-mediated signal network promotes prostate cancer metastatic colonization. *Endocr Relat Cancer* **2014**, *21*, 311-326, doi:10.1530/ERC-13-0548.
Prostate	Smith M.R. *et al.* Denosumab and bone metastasis-free survival in men with nonmetastatic castration-resistant prostate cancer: exploratory analyses by baseline prostate-specific antigen doubling time. *J Clin Oncol* **2013**, *31*, 3800-3806, doi:10.1200/JCO.2012.44.6716.
Prostate	Uehara H., Takahashi, T. and Izumi, K. Induction of retinol-binding protein 4 and placenta-specific 8 expression in human prostate cancer cells remaining in bone following osteolytic tumor growth inhibition by osteoprotegerin. *Int J Oncol* **2013**, *43*, 365-374, doi:10.3892/ijo.2013.1954.
Prostate	Smith M.R. *et al.* Denosumab and bone-metastasis-free survival in men with castration-resistant prostate cancer: results of a phase 3, randomised, placebo-controlled trial. *Lancet* **2012**, *379*, 39-46, doi:10.1016/S0140-6736(11)61226-9.
Prostate	Dai J., Lu, Y., Yu, C., Keller, J.M., Mizokami, A., Zhang, J. and Keller, E.T. Reversal of chemotherapy-induced leukopenia using granulocyte macrophage colony-stimulating factor promotes bone metastasis that can be blocked with osteoclast inhibitors. *Cancer Res* **2010**, *70*, 5014-5023, doi:10.1158/0008-5472.CAN-10-0100.
Prostate	Virk M.S. *et al.* Influence of simultaneous targeting of the bone morphogenetic protein pathway and RANK/RANKL axis in osteolytic prostate cancer lesion in bone. *Bone* **2009**, *44*, 160-167, doi:10.1016/j.bone.2008.09.009.
Prostate	Miller R.E. *et al.* RANK ligand inhibition plus docetaxel improves survival and reduces tumor burden in a murine model of prostate cancer bone metastasis. *Mol Cancer Ther* **2008**, *7*, 2160-2169, doi:10.1158/1535-7163.MCT-08-0046.
Prostate	Ignatoski K.M. *et al.* RANKL inhibition is an effective adjuvant for docetaxel in a prostate cancer bone metastases model. *Prostate* **2008**, *68*, 820-829, doi:10.1002/pros.20744.
Prostate	Armstrong A.P. *et al.* RANKL acts directly on RANK-expressing prostate tumor cells and mediates migration and expression of tumor metastasis genes. *Prostate* **2008**, *68*, 92-104, doi:10.1002/pros.20678.

	Skin	Asano T., Okamoto, K., Nakai, Y., Tsutsumi, M., Muro, R., Suematsu, A., Hashimoto, K., Okamura, T., Ehata, S., Nitta, T. and Takayanagi, H. Soluble RANKL is physiologically dispensable but accelerates tumour metastasis to bone. *Nat Metab* **2019**, *1*, 868-875, doi:10.1038/s42255-019-0104-1.
	Skin	Angela Y. *et al.* Combination of denosumab and immune checkpoint inhibition: experience in 29 patients with metastatic melanoma and bone metastases. *Cancer Immunol Immunother* **2019**, *68*, 1187-1194, doi:10.1007/s00262-019-02353-5.
	Skin	Jones D.H. *et al.* Regulation of cancer cell migration and bone metastasis by RANKL. *Nature* **2006**, *440*, 692-696, doi:10.1038/nature04524.
	Vulva	Canon J., Bryant, R., Roudier, M., Osgood, T., Jones, J., Miller, R., Coxon, A., Radinsky, R. and Dougall, W.C. Inhibition of RANKL increases the anti-tumor effect of the EGFR inhibitor panitumumab in a murine model of bone metastasis. *Bone* **2010**, *46*, 1613-1619, doi:10.1016/j.bone.2010.03.001.
**SCF**	Breast	Das Roy L. *et al.* Arthritis augments breast cancer metastasis: role of mast cells and SCF/c-Kit signaling. *Breast Cancer Res* **2013**, *15*, R32, doi:10.1186/bcr3412.
	Lung	Xiao H. *et al.* The release of tryptase from mast cells promote tumor cell metastasis via exosomes. *BMC Cancer* **2019**, *19*, 1015, doi:10.1186/s12885-019-6203-2.
**TGFB**	Breast	Meenu M. *et al.* Association of Monoamine Oxidase A with Tumor Burden and Castration Resistance in Prostate Cancer. *Curr Ther Res Clin Exp* **2020**, *93*, 100610, doi:10.1016/j.curtheres.2020.100610.
	Breast	Ma J. *et al.* WSZG inhibits BMSC-induced EMT and bone metastasis in breast cancer by regulating TGF-beta1/Smads signaling. *Biomed Pharmacother* **2020**, *121*, 109617, doi:10.1016/j.biopha.2019.109617.
	Breast	Futakuchi M. *et al.* The Effects of TGF-beta Signaling on Cancer Cells and Cancer Stem Cells in the Bone Microenvironment. *Int J Mol Sci* **2019**, *20*, doi:10.3390/ijms20205117.
	Breast	Di L. *et al.* Discovery of a natural small-molecule compound that suppresses tumor EMT, stemness and metastasis by inhibiting TGFbeta/BMP signaling in triple-negative breast cancer. *J Exp Clin Cancer Res* **2019**, *38*, 134, doi:10.1186/s13046-019-1130-2.
	Breast	Haley H.R. *et al.* Enhanced Bone Metastases in Skeletally Immature Mice. *Tomography* **2018**, *4*, 84-93, doi:10.18383/j.tom.2018.00010.
	Breast	Buenrostro D. *et al.* Early TGF-beta inhibition in mice reduces the incidence of breast cancer induced bone disease in a myeloid dependent manner. *Bone* **2018**, *113*, 77-88, doi:10.1016/j.bone.2018.05.008.
	Breast	Zhuang X. *et al.* Differential effects on lung and bone metastasis of breast cancer by Wnt signalling inhibitor DKK1. *Nat Cell Biol* **2017**, *19*, 1274-1285, doi:10.1038/ncb3613.
	Breast	Meng X., Vander Ark, A., Lee, P., Hostetter, G., Bhowmick, N.A., Matrisian, L.M., Williams, B.O., Miranti, C.K. and Li, X. Myeloid-specific TGF-beta signaling in bone promotes basic-FGF and breast cancer bone metastasis. *Oncogene* **2016**, *35*, 2370-2378, doi:10.1038/onc.2015.297.
	Breast	Xu Q. *et al.* Peripheral TGF-beta1 signaling is a critical event in bone cancer-induced hyperalgesia in rodents. *J Neurosci* **2013**, *33*, 19099-19111, doi:10.1523/JNEUROSCI.4852-12.2013.
	Breast	Rana T. *et al.* Doxorubicin-mediated bone loss in breast cancer bone metastases is driven by an interplay between oxidative stress and induction of TGFbeta. *PLoS One* **2013**, *8*, e78043, doi:10.1371/journal.pone.0078043.

Breast	Zhang Z. *et al.* Intravenous administration of adenoviruses targeting transforming growth factor beta signaling inhibits established bone metastases in 4T1 mouse mammary tumor model in an immunocompetent syngeneic host. *Cancer Gene Ther* **2012**, *19*, 630-636, doi:10.1038/cgt.2012.41.
Breast	Biswas S. *et al.* Anti-transforming growth factor ss antibody treatment rescues bone loss and prevents breast cancer metastasis to bone. *PLoS One* **2011**, *6*, e27090, doi:10.1371/journal.pone.0027090.
Breast	Ganapathy V. *et al.* Targeting the Transforming Growth Factor-beta pathway inhibits human basal-like breast cancer metastasis. *Mol Cancer* **2010**, *9*, 122, doi:10.1186/1476-4598-9-122.
Breast	Bandyopadhyay A. *et al.* Doxorubicin in combination with a small TGFbeta inhibitor: a potential novel therapy for metastatic breast cancer in mouse models. *PLoS One* **2010**, *5*, e10365, doi:10.1371/journal.pone.0010365.
Breast	Petersen M. *et al.* Smad2 and Smad3 have opposing roles in breast cancer bone metastasis by differentially affecting tumor angiogenesis. *Oncogene* **2010**, *29*, 1351-1361, doi:10.1038/onc.2009.426.
Breast	Dunn L.K. *et al.* Hypoxia and TGF-beta drive breast cancer bone metastases through parallel signaling pathways in tumor cells and the bone microenvironment. *PLoS One* **2009**, *4*, e6896, doi:10.1371/journal.pone.0006896.
Breast	Wilson T.J. *et al.* Cathepsin G-mediated enhanced TGF-beta signaling promotes angiogenesis via upregulation of VEGF and MCP-1. *Cancer Lett* **2010**, *288*, 162-169, doi:10.1016/j.canlet.2009.06.035.
Breast	Korpal M. *et al.* Imaging transforming growth factor-beta signaling dynamics and therapeutic response in breast cancer bone metastasis. *Nat Med* **2009**, *15*, 960-966, doi:10.1038/nm.1943.
Breast	Futakuchi M., Nannuru, K.C., Varney, M.L., Sadanandam, A., Nakao, K., Asai, K., Shirai, T., Sato, S.Y. and Singh, R.K. Transforming growth factor-beta signaling at the tumor-bone interface promotes mammary tumor growth and osteoclast activation. *Cancer Sci* **2009**, *100*, 71-81, doi:10.1111/j.1349-7006.2008.01012.x.
Breast	Deckers M. *et al.* The tumor suppressor Smad4 is required for transforming growth factor beta-induced epithelial to mesenchymal transition and bone metastasis of breast cancer cells. *Cancer Res* **2006**, *66*, 2202-2209, doi:10.1158/0008-5472.CAN-05-3560.
Breast	Hiraga T. *et al.* Stimulation of cyclooxygenase-2 expression by bone-derived transforming growth factor-beta enhances bone metastases in breast cancer. *Cancer Res* **2006**, *66*, 2067-2073, doi:10.1158/0008-5472.CAN-05-2012.
Breast	Kang Y. *et al.* Breast cancer bone metastasis mediated by the Smad tumor suppressor pathway. *Proc Natl Acad Sci U S A* **2005**, *102*, 13909-13914, doi:10.1073/pnas.0506517102.
Breast	Kakonen S.M., Selander, K.S., Chirgwin, J.M., Yin, J.J., Burns, S., Rankin, W.A., Grubbs, B.G., Dallas, M., Cui, Y. and Guise, T.A. Transforming growth factor-beta stimulates parathyroid hormone-related protein and osteolytic metastases via Smad and mitogen-activated protein kinase signaling pathways. *J Biol Chem* **2002**, *277*, 24571-24578, doi:10.1074/jbc.M202561200.
Breast	Yin J.J., Selander, K., Chirgwin, J.M., Dallas, M., Grubbs, B.G., Wieser, R., Massague, J., Mundy, G.R. and Guise, T.A. TGF-beta signaling blockade inhibits PTHrP secretion by breast cancer cells and bone metastases development. *J Clin Invest* **1999**, *103*, 197-206, doi:10.1172/JCI3523.
Kidney	Kominsky S.L. *et al.* TGF-beta promotes the establishment of renal cell carcinoma bone metastasis. *J Bone Miner Res* **2007**, *22*, 37-44, doi:10.1359/jbmr.061005.

	Lung	Yang X.Y. *et al.* FMNL1 down-regulation suppresses bone metastasis through reducing TGF-beta1 expression in non-small cell lung cancer (NSCLC). *Biomed Pharmacother* **2019**, *117*, 109126, doi:10.1016/j.biopha.2019.109126.	
	Prostate	Lang C. *et al.* SMAD3/SP1 complex-mediated constitutive active loop between lncRNA PCAT7 and TGF-beta signaling promotes prostate cancer bone metastasis. *Mol Oncol* **2020**, *14*, 808-828, doi:10.1002/1878-0261.12634.	
	Prostate	Dai Y. *et al.* Copy number gain of ZEB1 mediates a double-negative feedback loop with miR-33a-5p that regulates EMT and bone metastasis of prostate cancer dependent on TGF-beta signaling. *Theranostics* **2019**, *9*, 6063-6079, doi:10.7150/thno.36735.	
	Prostate	Meng X., Vander Ark, A., Daft, P., Woodford, E., Wang, J., Madaj, Z. and Li, X. Loss of TGF-beta signaling in osteoblasts increases basic-FGF and promotes prostate cancer bone metastasis. *Cancer Lett* **2018**, *418*, 109-118, doi:10.1016/j.canlet.2018.01.018.	
	Prostate	TGFbeta Induces a Pro-Bone Metastasis Program in Prostate Cancer. *Cancer Discov* **2015**, *5*, OF23, doi:10.1158/2159-8290.CD-RW2015-098.	
	Prostate	Fournier P.G. *et al.* The TGF-beta Signaling Regulator PMEPA1 Suppresses Prostate Cancer Metastases to Bone. *Cancer Cell* **2015**, *27*, 809-821, doi:10.1016/j.ccell.2015.04.009.	
	Prostate	Hu Z. *et al.* Systemic delivery of oncolytic adenoviruses targeting transforming growth factor-beta inhibits established bone metastasis in a prostate cancer mouse model. *Hum Gene Ther* **2012**, *23*, 871-882, doi:10.1089/hum.2012.040.	
	Prostate	Wan X. *et al.* Effect of transforming growth factor beta (TGF-beta) receptor I kinase inhibitor on prostate cancer bone growth. *Bone* **2012**, *50*, 695-703, doi:10.1016/j.bone.2011.11.022.	
	Prostate	Mishra S. *et al.* Blockade of transforming growth factor-beta (TGFbeta) signaling inhibits osteoblastic tumorigenesis by a novel human prostate cancer cell line. *Prostate* **2011**, *71*, 1441-1454, doi:10.1002/pros.21361.	
	Skin	Bandyopadhyay A. *et al.* Inhibition of pulmonary and skeletal metastasis by a transforming growth factor-beta type I receptor kinase inhibitor. *Cancer Res* **2006**, *66*, 6714-6721, doi:10.1158/0008-5472.CAN-05-3565.	Refers to spine metastases
	Skin	Javelaud D. *et al.* Stable overexpression of Smad7 in human melanoma cells impairs bone metastasis. *Cancer Res* **2007**, *67*, 2317-2324, doi:10.1158/0008-5472.CAN-06-3950.	
**TGFB1**	Oral cavity	Davies M. *et al.* Endogenous TGF-beta1 inhibits the growth and metastatic dissemination of rat oral carcinoma cell lines but enhances local bone resorption. *J Oral Pathol Med* **2000**, *29*, 232-240, doi:10.1034/j.1600-0714.2000.290507.x.	
**TNFA**	Breast	Bishop R.T. *et al.* Combined administration of a small-molecule inhibitor of TRAF6 and Docetaxel reduces breast cancer skeletal metastasis and osteolysis. *Cancer Lett* **2020**, *488*, 27-39, doi:10.1016/j.canlet.2020.05.021.	
	Breast	Lei W., Duan, R., Li, J., Liu, X., Huston, A., Boyce, B.F. and Yao, Z. The IAP Antagonist SM-164 Eliminates Triple-Negative Breast Cancer Metastasis to Bone and Lung in Mice. *Sci Rep* **2020**, *10*, 7004, doi:10.1038/s41598-020-64018-z.	
	Breast	Hamaguchi T. *et al.* TNF inhibitor suppresses bone metastasis in a breast cancer cell line. *Biochem Biophys Res Commun* **2011**, *407*, 525-530, doi:10.1016/j.bbrc.2011.03.051.	
	Lung	Young M.R. and Wright, M.A. Myelopoiesis-associated immune suppressor cells in mice bearing metastatic Lewis lung carcinoma tumors: gamma interferon plus tumor necrosis factor alpha synergistically reduces immune suppressor and	

		tumor growth-promoting activities of bone marrow cells and diminishes tumor recurrence and metastasis. *Cancer Res* **1992**, *52*, 6335-6340, .
**TNFSF14**	Lung	Brunetti G., Belisario, D.C., Bortolotti, S., Storlino, G., Colaianni, G., Faienza, M.F., Sanesi, L., Alliod, V., Buffoni, L., Centini, E., Voena, C., Pulito, R., Novello, S., Ingravallo, G., Rizzi, R., Mori, G., Reseland, J.E., Ware, C.F., Colucci, S., Ferracini, R., Grano, M. and Roato, I. LIGHT/TNFSF14 Promotes Osteolytic Bone Metastases in Non-small Cell Lung Cancer Patients. *J Bone Miner Res* **2020**, *35*, 671-680, doi:10.1002/jbmr.3942.
**TRAIL**	Breast	Holland P.M., Miller, R., Jones, J., Douangpanya, H., Piasecki, J., Roudier, M. and Dougall, W.C. Combined therapy with the RANKL inhibitor RANK-Fc and rhApo2L/TRAIL/dulanermin reduces bone lesions and skeletal tumor burden in a model of breast cancer skeletal metastasis. *Cancer Biol Ther* **2010**, *9*, 539-550, doi:10.4161/cbt.9.7.11266.
**TRAILR2**	Breast	Fritsche H. *et al.* TRAIL-R2 promotes skeletal metastasis in a breast cancer xenograft mouse model. *Oncotarget* **2015**, *6*, 9502-9516, doi:10.18632/oncotarget.3321.
**VEGF**	Breast	Di Benedetto M. *et al.* MDA-MB-231 breast cancer cells overexpressing single VEGF isoforms display distinct colonisation characteristics. *Br J Cancer* **2015**, *113*, 773-785, doi:10.1038/bjc.2015.267.
**VEGFR**	Lung	Fujita H., Gomori, A., Fujioka, Y., Kataoka, Y., Tanaka, K., Hashimoto, A., Suzuki, T., Ito, K., Haruma, T., Yamamoto-Yokoi, H., Harada, N., Sakuragi, M., Oda, N., Matsuo, K., Inada, M. and Yonekura, K. High Potency VEGFRs/MET/FMS Triple Blockade by TAS-115 Concomitantly Suppresses Tumor Progression and Bone Destruction in Tumor-Induced Bone Disease Model with Lung Carcinoma Cells. *PLoS One* **2016**, *11*, e0164830, doi:10.1371/journal.pone.0164830.
